# Digital vs. Direct Anthropometry with MetiSmile^®^ 3D Face Scanner: A Validation and Reliability Study on a Mannequin Model

**DOI:** 10.3390/cmtr19010003

**Published:** 2025-12-30

**Authors:** Alexander De Crem, Constantijn Bogaert, Frederik Piccart, Matthias Ureel, Benjamin Denoiseux, Lisa De Kock, Marieke Brands, Olivier Lenssen, Renaat Coopman

**Affiliations:** 1 Department of Oral and Maxillofacial Surgery and Head and Neck Surgery, Vrije Universiteit Brussel (VUB), 1090 Brussels, Belgium; alexander.decrem@uzbrussel.be (A.D.C.); matthias.ureel@uzgent.be (M.U.); benjamin.denoiseux@uzgent.be (B.D.); lisa.dekock@uzgent.be (L.D.K.); 2 Department of Oral and Craniomaxillofacial (OCMF) Surgery, University Hospital Ghent, 9000 Ghent, Belgium; constantijn.bogaert@ugent.be (C.B.); marieke.brands@uzbrussel.be (M.B.); olivier.lenssen@uzbrussel.be (O.L.); 3 Ziekenhuis Heusden-Zolder, 3550 Heusden-Zolder, Belgium; frederik.piccart@sfz.be; 4 Cancer Research Institute Ghent (CRIG), 9000 Ghent, Belgium; 5 Sint-Blasius Hospital, 9200 Dendermonde, Belgium; 6 Department of Oral and Craniomaxillofacial (OCMF) Surgery, University Hospital Brussels (UZ Brussel), 1090 Brussels, Belgium; 7 Department of Oral and Craniomaxillofacial (OCMF) Surgery, Ziekenhuis Netwerk Antwerpen (ZNA), Middelheim Hospital, 2020 Antwerp, Belgium; 8 Integraal Kankercentrum Nederland (IKNL), 3511 LC Utrecht, The Netherlands

**Keywords:** facial scan, facial anthropometry, orthognathic surgery, digitalization, facial analysis

## Abstract

Background: Three-dimensional facial anthropometry is increasingly used in orthodontics and orthognathic surgery. Conventional face scanning systems such as Vectra^®^ and 3dMD^®^ are well validated but remain costly and technically demanding. The MetiSmile^®^ 3D face scanner provides a more affordable and portable alternative, yet its accuracy and reproducibility have not been rigorously evaluated. Methods: Validation was performed on a standardized mannequin head in two phases. Phase 1 assessed mesh reproducibility under artificial lighting (AL), natural lighting (n-AL), and after mesh-refinement (AL-F). Landmark-based pre-registration with Iterative Closest Point refinement was applied; root-mean-square error (RMS) and distance maps were calculated. In phase 2, three observers (student, resident, consultant) performed 14 predefined linear measurements by direct anthropometry (DA) and digital anthropometry (DiA). Intra- and inter-observer reliability was evaluated using intraclass correlation coefficients. Results: Phase 1 yielded mean RMS values of 0.041 mm (AL), 0.043 mm (n-AL), and 0.030 mm (AL-F), with largest deviations near eyes, alar regions, and lip commissures. Phase 2 showed excellent ICCs (≥0.997) and mean absolute DA–DiA differences of 0.25–0.33 mm, with only few differences > 2 mm. Conclusion: The MetiSmile^®^ scanner generates highly reproducible meshes and clinically acceptable linear measurements on mannequin models. Further validation on live subjects is warranted before routine clinical application.

## 1. Introduction

In the diagnostic work-up for orthognathic procedures, facial anthropometry plays a central role. The quantitative assessment of facial dimensions and proportions was traditionally performed using direct anthropometry which involved the manual use of calipers and rulers. This has long been considered the gold standard due to its simplicity and direct applicability [1,2,3,4].

As in many other disciplines, digitization is transforming OCMF diagnostics and treatment planning. A key advantage lies in the acquisition and analysis of complete 3D datasets, which provide more comprehensive and reproducible measurements. In contrast to 2D photography, 3D imaging enables realistic simulation, longitudinal monitoring, and seamless integration with other digital modalities (e.g., intraoral scans, CBCT) [5]. This has led to a shift towards digital anthropometry, which leverages 3D imaging and computational analysis to measure and evaluate facial structures.

A variety of 3D imaging methods and commercial systems are currently available for digital facial anthropometry. These include stereophotogrammetry, structured light scanning, photogrammetry, laser surface scanning, and time-of-flight (ToF) systems. Each technology offers clinically acceptable results and carries its own set of strengths and limitations [6].

The MetiSmile^®^ scanner (Shining3D, Hangzhou, China) represents a compact and portable stereophotogrammetric system designed for soft tissue analysis in dentistry and OCMF surgery. It combines four calibrated cameras with an infrared proximity sensor, facilitating rapid acquisition of facial geometry without the use of visible flash. While the MetiSmile^®^ system shows promising potential for clinical integration, formal validation studies are still required to establish its accuracy, reliability, and clinical usability.

This study therefore aims to validate the MetiSmile^®^ 3D facial scanner as a stepping stone toward establishing a validated, fully digital and clinically acceptable 3D anthropometric workflow for orthognathic surgery consultations.

## 2. Materials and Methods

### 2.1. Study Design

The validation of the MetiSmile^®^ 3D facial scanner was conducted in two phases. Phase I focused on reproducibility and precision by analyzing the consistency of repeated scans under varying lighting conditions and across different operators. Phase II addressed accuracy by comparing digital anthropometric measurements obtained with the MetiSmile^®^ system (Digital Anthropometry, DiA) against traditional direct anthropometry (DA) using calipers on the same reference model.

Across both phases, three fundamental quality parameters were assessed: accuracy, precision, and reproducibility. Together, these metrics determine the clinical applicability of the scanning system [7,8,9].

Accuracy (Trueness) describes how closely measurements correspond to a reference or “gold standard.” It is typically assessed by comparing 3D models from the test device with high-resolution reference scans or certified phantoms (e.g., plaster casts, 3D-printed replicas, anthropometric models). Deviations are quantified with metrics such as Root Mean Square (RMS) Error or Mean Absolute Error (MAE).

Precision (Repeatability) reflects the consistency of repeated measurements under identical conditions. This is evaluated by performing multiple scans of the same object without altering settings, followed by inter-comparison of models. Variability is expressed using the Intraclass Correlation Coefficient (ICC), Coefficient of Variation (CV), or standard deviation of differences.

Reproducibility (Inter-Observer Reliability) indicates robustness under varying conditions or operators. It is tested by repeating scans across different users or settings, with agreement assessed through ICC values and Bland–Altman plots. High reproducibility confirms the system’s reliability in clinical workflows.

### 2.2. Reference Model and Anatomical Landmarks

**Figure 1:**
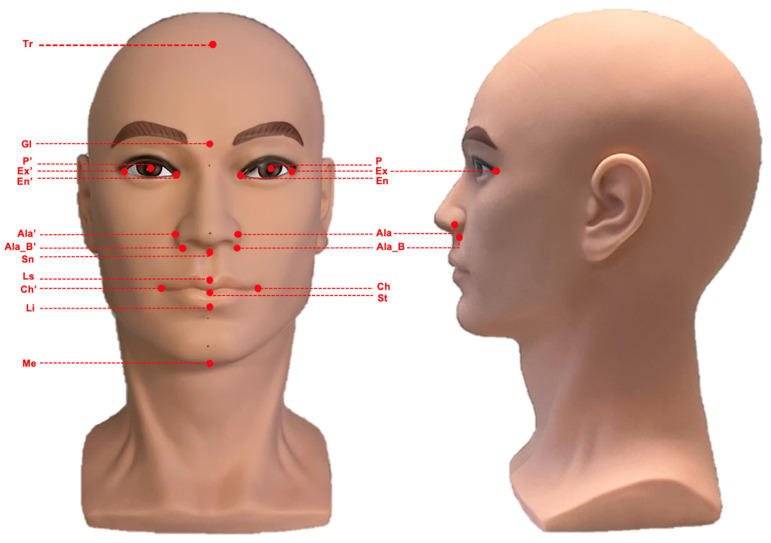
Standardized mannequin head used for three-dimensional facial imaging and measurement analyses. The image shows the frontal (**left**) and lateral (**right**) views. Abbreviations: Trichion (Tr), Glabella (Gl), Subnasale (Sn), Labiale Superior (Ls), Stomion (St), Labiale inferior (Li), Menton (Me), Cheilon (Ch/Ch’), Ala (Ala/Ala’), Alar base (Ala_B/Ala_B’), Exocanthion (Ex/Ex’), Endocanthion (En/En’), Center of pupil (P/P’).

A standardized mannequin head (bald male, soft PVC, head circumference 55.90 cm) served as the surrogate model throughout the study (Figure 1). Nineteen anatomical landmarks were marked on the facial surface using a fine black marker (Artline 200 Fine 0.4) by the primary author and verified by the senior author [10]. These landmarks were selected to represent the vertical upper, middle, and lower thirds of the face, ensuring appropriate coverage of key facial regions for linear measurement. From these landmarks, 14 linear measurements were defined (Table 1).

Direct anthropometric measurements were obtained with a digital caliper (Fiber Composite Digital Caliper—resolution: 0.100 mm; accuracy: 0.200 mm) served as the gold standard for accuracy analyses. Digital scans were acquired using the MetiSmile^®^ scanner and analyzed with the Shining3D software packages version 2.2.5.7 (FScan, Shining3D, Hangzhou, China; Facial Analyzer, Shining3D, Hangzhou, China). All processing was performed on a Dell Precision 7680 workstation (Intel 13th generation i7-13850HX processor, 2 × 16 GB RAM, Windows 11).

#### 2.2.1. Phase I: Mesh Reproducibility and Precision Analysis

**Figure 2:**
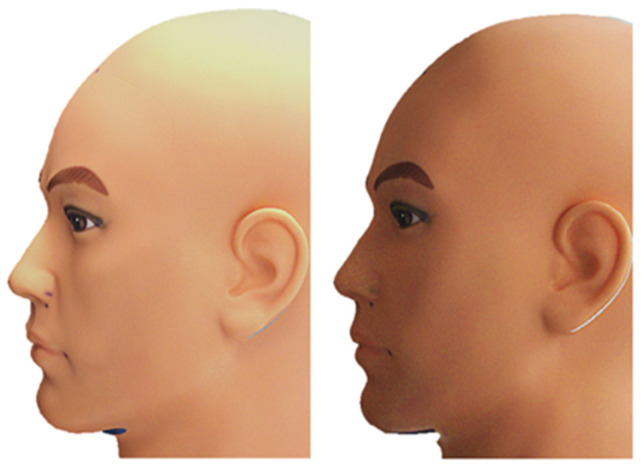
Left: AL Scan n°3; Right: AL-F Scan n°3.

To evaluate the reproducibility of mesh acquisition, the mannequin was scanned 20 times by the main researcher under two different lighting conditions. Ten scans were performed with ambient artificial lighting combined with daylight from a window (AL scans), while the remaining ten were carried out in the evening under overcast conditions without artificial light (n-AL scans). In both settings, the brightness of the scanner was automatically optimized by the software. The resulting meshes were cropped to include the face, ears and part of the neck. (Figure 2) One mesh (n°3) was randomly selected as the reference mesh, to which all other meshes were aligned using three common anatomical landmarks (exocanthion left, exocanthion right and pronasale) followed by fine registration with Iterative Closest Point (ICP) alignment.

For clinical evaluation, the artificially lit meshes were subsequently trimmed to include the facial regions of highest relevance. The trimming was performed in a circular fashion as to include all facial landmarks anterior of the trichion, tragal point and cervical point.

#### 2.2.2. Phase II: Accuracy—Direct Anthropometry vs. Digital Anthropometry

For the assessment of accuracy, both DA and DiA were performed on the mannequin, that consisted of a fixed head component mounted on an integrated stand. For each measurement, the entire assembly was placed on the same flat table surface at eye level of the primary investigator to ensure a consistent baseline position. On this stable platform, the head was visually aligned to the Frankfort horizontal plane by checking the tragion–orbitale line in the frontal and lateral views. Because the head remained in the same fixed position throughout all scans, a consistent orientation across consecutive acquisitions was guaranteed.

During scanning, the mannequin remained completely static while the examiner moved the handheld MetiSmile^®^ scanner (FScan, Shining3D, Hangzhou, China; Facial Analyzer, Shining3D, Hangzhou, China). around the facial surface in accordance with the manufacturer’s recommended workflow. This involved a continuous sweep along the frontal, lateral, and inferior facial regions. The average time required to complete a full scan was approximately 15–20 s, depending on operator experience.

The measurements were carried out by three observers with different levels of experience: a dental student (DS) with no prior experience in anthropometry, a maxillofacial surgery trainee (MT) with eight years of experience, and a maxillofacial surgery consultant (MC) with more than ten years of experience. Each observer performed three repeated measurements per DA and DiA session, yielding a total of six measurements for both methods. To further reduce the influence of memory, a second series of sessions was repeated after two weeks.

DA measurements were performed with a 150 mm digital caliper (Carbon Fiber Composite Digital Caliper, resolution: 0.100 mm, accuracy: ±0.200 mm). DiA measurements were obtained from 3D scans generated with the MetiSmile^®^ system. Semi-automated landmark detection was provided by the Shining3D Face Analyzer software 2.2.5.7., although manual repositioning was required to ensure alignment with the predefined 19 landmarks. For four linear distances were not automatically generated (see table) and had to be measured manually through the “Free Measure” tool which allows measuring distances between two (freely) chosen points.

### 2.3. Statistical Analysis

In Phase I, reproducibility was evaluated by comparing meshes through the calculation using CloudCompare v2.13 beta of RMS error, mean deviations, and 95% confidence intervals to quantify intra-device variability under different lighting conditions (Table 2) [11].

In Phase II, which focused on accuracy and inter-observer reliability, descriptive statistics including range, interquartile range (IQR), mean, and difference in means were generated in IBM SPSS Statistics 26 after collecting both DA and DiA measurements. Because of the limited sample size and non-normally distributed data, we opted to use the median and IQR as robust, non-parametric measures of variability instead of the standard deviation. Negative deviation values indicate that the DiA measurement exceeded the corresponding DA measurement. To assess intra- and inter-observer reliability, a two-way mixed-effects model with absolute agreement was used, and single-measure ICC values were reported. For inter-observer reliability specifically, ICCs (two-way mixed, average measures) were calculated for both the direct and digital measurements between each pair of observers [8]. Separate ICC values were obtained for DA and DiA, as well as for the combined dataset. In addition, Bland–Altman plots were generated to provide a visual assessment of agreement and bias between observers and across measurement methods [7,9]. RMS values (mean, minimum, and maximum) are reported as positive values, reflecting the absolute magnitude of measurement error.

## 3. Results

### 3.1. Mesh Reproducibility

**Figure 3:**
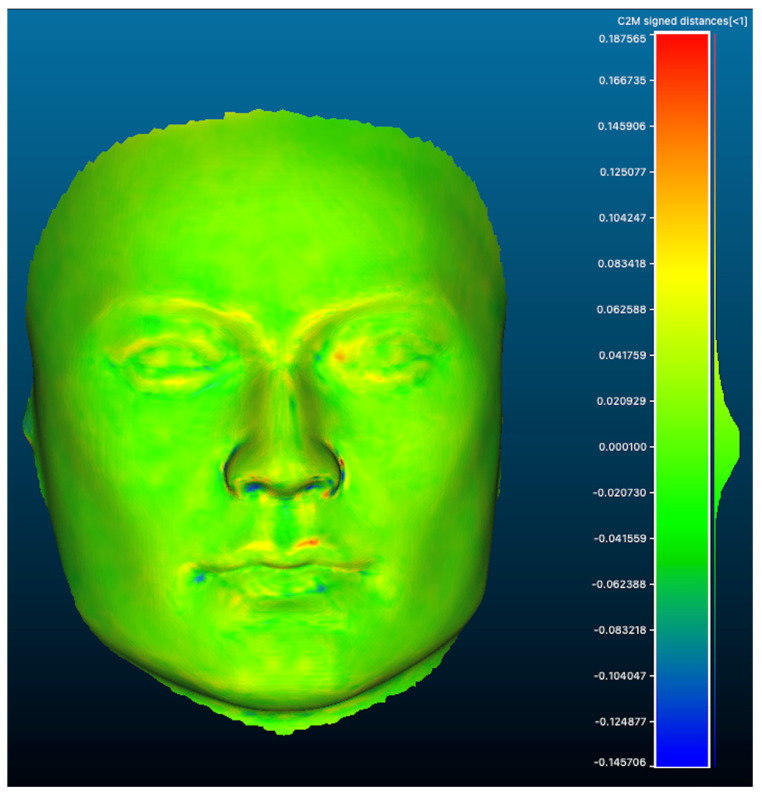
Colormap of distances between meshes (scan AL-F n°5 vs reference mesh) showing a narrow distribution with largest distances seen as a red (+) or blue (−) color (highest distance).

**Figure 4:**
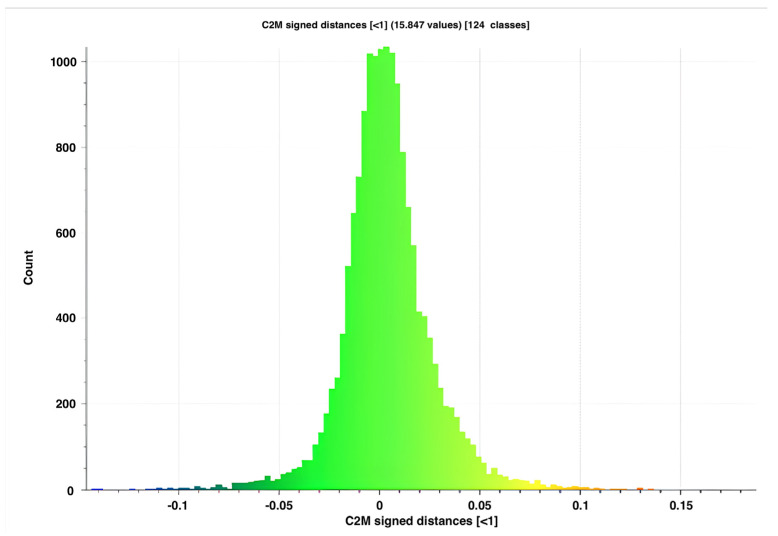
Distribution of distances for AL-F scan n°5 compared to the reference mesh.

The AL-scans showed a maximal and minimal deviation of, respectively, 0.345 and −0.414 mm (range: 0.759 mm) when aligned with the reference scan. The n-AL scans showed a smaller range with a maximal and minimal distance of, respectively, 0.351 and −0.339 mm (range: 0.690 mm). The scans edited to contain the clinical region of interest only (AL-F scans) had maximal and minimal deviations of, respectively, 0.252 and −0.191 mm (range: 0.443). The highest distances between meshes were seen in the inferior and inferiolateral nasal region, auricle, Glogau-Klein points (upper cupid’s bow), cheilon, endo- and exocanthion, submental point, medial half of the supraorbital margin, upper and lower eyelid margins and submental region (see Figure 3 distance color map). The final RMS for the AL, n-AL and AL-F scans was 0.041, 0.043 and 0.030 with the mean distances, respectively, being −0.002 mm, −0.004 mm and 0.003 mm and submillimeter confidence intervals (Figure 4: narrow distribution of distances).

### 3.2. Inter- and Intra-Observer Reliability

The descriptive analyses show a range for direct and digital measurements of, respectively, 1.610 and 1.900 mm for the MC, 0.940 and 1.090 mm for the MT, and 0.670 and 1.430 mm for the DS. The difference in means between the direct and digital measurements were 0.250 mm for the consultant, 0.330 mm for the trainee and 0.320 mm for the student (Table 3).

When analyzing the ICC, excellent intra-observer reliability (ICC > 0.900) is noted for all observers independent for both the direct and the digital anthropometry methods for each observer. Excellent inter-observer reliability is noted as well between each pair of observers for both the direct as well as the digital measuring method. (Table 4 and Table 5) The same results were seen for the inter-observer reliability between all three observers for both direct and digital anthropometry.

**Figure 5:**
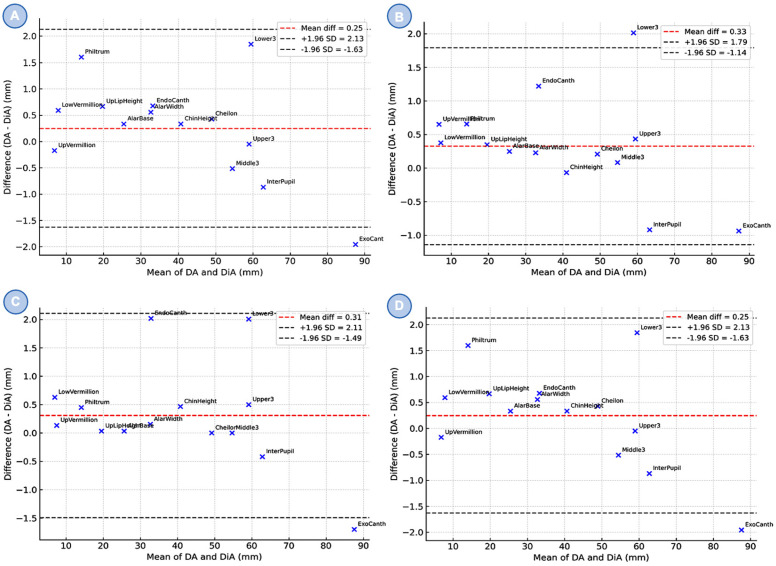
Bland–Altman plots comparing direct anthropometry (DA) and digital anthropometry (DiA) across three groups: (**A**) maxillofacial surgery consultant (MC), (**B**) maxillofacial surgery trainee (MT), (**C**) dental student (DS) and combined results (**D**). The red dashed line indicates the mean difference between methods, while the gray dashed lines represent the 95% limits of agreement (±1.96 SD). Each dot corresponds to a specific anatomical landmark.

Bland–Altman plots (Figure 5) demonstrated strong agreement between DA and DiA across all observers. The consultant group showed a mean difference of 0.25 mm (95% LoA −1.630 to +2.130 mm), the trainee 0.330 mm (−1.140 to +1.790 mm), and the student 0.310 mm (−1.490 to +2.110 mm). The combined dataset confirmed a consistent submillimeter bias with narrow limits of agreement (LoA). Individual anatomical landmarks were evenly distributed without evidence of systematic measurement bias.

## 4. Discussion

Stereophotogrammetry remains one of the most reliable and established modalities for 3D facial imaging [1,2]. Systems such as Vectra (Canfield Scientific), 3dMDface (3dMD), and Di3D (Dimensional Imaging) acquire simultaneous images from multiple fixed cameras and reconstruct facial geometry through triangulation, offering high surface accuracy and suitability for a wide range of clinical applications [12,13]. Structured light scanning represents an alternative approach in which a projected pattern is distorted across the facial surface and captured by cameras to compute depth. Examples include the Artec Eva (Artec 3D), Bellus3D Face Camera Pro, and HP Structured Light Scanner. These systems are valued for their high resolution and are used in prosthodontics, surgical planning, and digital smile design.

The choice of imaging modality should be guided by clinical requirements, including resolution, workflow integration, patient comfort, and cost. Regardless of the system selected, rigorous validation is essential before clinical implementation.

The MetiSmile^®^ handheld structured light scanner is a recent addition to this field. Several groups have investigated its performance. Bor et al. and Nuytens et al. demonstrated in vitro that MetiSmile^®^ outperformed desktop and mobile scanners in both trueness (0.18 ± 0.15 mm) and precision (0.22 ± 0.04 mm), even surpassing repeated anthropometric caliper measurements [14,15]. A subsequent study by the same group confirmed that MetiSmile^®^ achieved the highest mesh density and regional accuracy, particularly in perioral regions, although acquisition required longer than with stationary systems [16]. Kumar et al. validated the scanner in vivo, showing the lowest mean deviation compared with CBCT (0.35 ± 0.33 mm) and superior accuracy relative to Carestream and Medit scanners [17]. Together with the present study, these reports provide converging evidence that MetiSmile^®^ consistently produces clinically acceptable—and often superior—results across diverse settings.

In the first phase of our study, reproducibility of meshes was confirmed across different lighting conditions. RMS values for artificial and natural light scans were almost identical (0.041 vs. 0.043 mm), indicating robustness to environmental variation. Restricting analysis to clinically relevant facial regions (AL-F meshes) further improved RMS values (0.030 mm) and reduced the deviation range by 41.6%. As in previous work, the largest errors occurred in anatomically complex regions such as the nasal base, eyelid margins, commissures, and periorbital areas (Figure 3) [18]. Compared with validated stereophotogrammetric and structured light systems, the MetiSmile^®^ results were equal or better [4,19,20,21,22,23,24]. However, mannequin-based RMS values cannot be directly extrapolated to live patients, where skin texture, soft tissue elasticity, and movement introduce additional complexity.

The second phase compared DA and DiA. While most landmarks were generated automatically, several clinically relevant distances (e.g., alar base width, lip height) required manual placement, reducing efficiency and reproducibility. Landmarks were sometimes difficult to visualize on the mesh, and no strict protocol was used for their annotation. Establishing standardized protocols and implementing customizable measurement sets in the software would improve consistency and clinical utility. Despite these limitations, intra- and inter-observer ICCs were excellent (≥0.997). Mean DA–DiA differences remained well below 1 mm, confirming clinical acceptability [1,25]. Only a few landmarks exceeded ±2 mm, mainly at anatomically complex sites such as the exocanthion, cheilon, and philtrum height. These discrepancies are likely related to anatomy and landmark placement rather than scanner limitations, but they highlight the need for improved protocols and automated landmark detection.

The time efficiency of digital measurements remains unclear. Unlike caliper-based methods, digital analysis involves scanner calibration, acquisition, and annotation. Future studies should directly compare workflow efficiency, particularly since some dental parameters (e.g., overjet, incisor display) still require direct measurement. Importantly, ICCs in this study were likely strengthened by the use of pre-marked landmarks on the mannequin, which may not fully extrapolate to clinical settings. Inter-observer variability in live patients should therefore be quantified in follow-up studies.

This study has several limitations that should be acknowledged. First, the use of a single standardized mannequin head inherently restricts the generalizability of the findings. This choice was deliberate, as working with one surrogate model allowed us to isolate measurement variability without introducing confounding morphological differences between specimens. Second, the anatomical landmarks were pre-marked on the mannequin head to ensure consistency across repeated measurements and to reduce landmark localization ambiguity. While we recognize that performing measurements on unmarked landmarks may better reflect real-world clinical practice, the controlled setup of this exploratory study enabled a focused and reliable comparison between direct and digital anthropometry. Future research should expand on this work by including multiple surrogate models and evaluating measurements based on unmarked anatomical landmarks to improve clinical applicability and external validity.

A strength of this work was the inclusion of multiple operators with varying expertise, yet excellent ICCs were maintained. This indicates robustness to user variation. However, a notable limitation is the exclusive use of a male mannequin head, which does not reflect sex-related differences in facial curvature and soft tissue distribution. Although current evidence suggests that scanner accuracy is unlikely to differ substantially between sexes, this limitation should be acknowledged. Furthermore, mannequin-based validation cannot account for patient-related factors such as motion, skin reflectivity, and heterogeneity in facial morphology.

Taken together, these considerations highlight the need for broader and more clinically oriented research. Several important avenues for future research arise from this work. Further studies are needed to evaluate newer 3D-scanning technologies, including stereophotogrammetry systems. In vivo validation on living subjects is also essential to account for soft-tissue dynamics, patient movement, and varying environmental conditions. Additionally, the role of proper calibration requires investigation, as inadequate calibration may introduce systematic errors. Research into inter- and intra-observer variability is equally important to quantify observer-dependent measurement differences. Such studies will provide crucial insight into potential sources of error. Together, these efforts will help improve the accuracy, precision, and reproducibility of facial measurements in orthognathic surgery.

## 5. Conclusions

In summary, this study adds to growing evidence that the MetiSmile^®^ scanner is accurate, reproducible, and clinically acceptable. While mannequin studies cannot replace patient validation, our findings—combined with recent reports—support MetiSmile^®^ as a reliable, cost-effective tool for orthodontic and orthognathic workflows. Future research should prioritize in vivo validation across diverse populations, both male and female, and explore integration with intraoral and CBCT data to move towards a validated and fully digital workflow. The development of automated landmark detection, standardized measurement protocols, and systematic evaluation of workflow efficiency will be essential for translating this technology into routine clinical practice.

## Figures and Tables

**Table 1:** Linear measurements.

Vertical Distances	Landmarks
Upper facial ⅓ *	Trichion, Glabella
Middle ⅓ *	Glabella, Subnasale
Lower ⅓	Subnasale, Menton
Upper lip height *	Subnasale, Stomion
Chin height	Stomion, Menton
Philtrum Length	Subnasale, Labiale Superior
Upper vermilion	Labiale Superior, Stomion
Lower vermilion	Stomion, Labiale Inferior
**Horizontal Distances**	**Landmarks**
Interpupillary distance	Center of Pupil Left and Right
Ca-Ca medial	Endocanthion Left and Right
Ca-Ca lateral	Exocanthion Left and Right
Alar width	Ala Left and Right
Alar base width *	Alar base Left and Right
Mouth Width (Ch-Ch)	Cheilon Left and Right

*: these measurements were not automatically generated in the FScan software version 2.2.5.7.

**Table 2:** Average distances compared with the reference mesh.

	Final RMS	Mean	95% C.I.	Minimal	Maximum
**AL scans**	0.041	−0.002	−0.097; 0.093	−0.414	0.345
**n-AL scans**	0.043	−0.004	−0.099; 0.091	−0.339	0.351
**AL-F scans**	0.030	0.003	−0.130; 0.136	−0.191	0.252

**Table 3:** Summary of the comparison between direct anthropometry (DA) and digital anthropometry (DiA) across three observers: maxillofacial surgery consultant (MC), maxillofacial surgery trainee (MT), and dental student (DS).

Group	Region	N	Range_DA	IQR_DA	Mean_DA	Mean_DiA	IQR_DiA	Range_DiA	Mean_DA-DiA
**MC**	**Upper3**	6vs6	2.000	0.800	58.933	58.983	0.830	1.200	−0.050
	**Middle3**	6vs6	1.000	0.470	54.216	54.733	0.600	0.900	−0.517
	**Lower3**	6vs6	3.700 *	1.750	60.400	58.557	1.580	2.640 *	1.843 *
	**InterPupil**	6vs6	0.500	0.280	62.350	63.218	0.660	1.900	−0.868
	**EndoCanth**	6vs6	2.200 *	1.220	33.567	32.888	3.630 *	4.750 *	0.678
	**ExoCanth**	6vs6	0.700	0.400	86.567	88.523	1.580	2.340 *	−1.957 *
	**AlarBase**	6vs6	0.800	0.730	25.583	25.250	0.320	0.400	0.333
	**AlarWidth**	6vs6	0.300	0.150	32.967	32.408	0.680	1.100	0.558
	**Cheilon**	6vs6	0.900	0.520	49.117	48.688	2.600 *	3.620 *	0.428
	**UpLipHeight**	6vs6	1.000	0.920	20.033	19.367	0.300	0.600	0.667
	**Philtrum**	6vs6	1.900	0.700	14.817	13.220	1.320	1.940	1.597 *
	**ChinHeight**	6vs6	3.600 *	1.950	40.767	40.433	0.630	1.000	0.333
	**UpVermillion**	6vs6	1.300	0.700	6.717	6.890	1.150	1.650	−0.173
	**LowVermillion**	6vs6	2.700 *	1.280	8.100	7.507	2.340 *	2.620 *	0.593
	***Mean:***		1.610					1.900	0.250
**MT**	**Upper3**	6vs6	1.000	0.770	59.717	59.283	0.220	0.300	0.433
	**Middle3**	6vs6	0.500	0.350	54.733	54.650	0.450	0.600	0.083
	**Lower3**	6vs6	1.800	1.130	59.950	57.935	1.150	2.720 *	2.015 *
	**InterPupil**	6vs6	0.800	0.350	62.833	63.752	0.700	0.950	−0.918
	**EndoCanth**	6vs6	0.900	0.380	34.083	32.862	0.930	1.610	1.222 *
	**ExoCanth**	6vs6	0.600	0.380	86.783	87.720	0.950	1.710	−0.937
	**AlarBase**	6vs6	0.400	0.180	25.750	25.500	0.580	1.100	0.250
	**AlarWidth**	6vs6	0.500	0.270	32.800	32.572	0.410	0.580	0.228
	**Cheilon**	6vs6	1.200	0.820	49.367	49.157	0.450	0.970	0.210
	**UpLipHeight**	6vs6	1.500	1.420	19.783	19.433	0.380	0.600	0.350
	**Philtrum**	6vs6	1.100	0.500	14.467	13.810	0.400	0.490	0.657
	**ChinHeight**	6vs6	0.700	0.550	40.900	40.967	0.380	0.900	−0.067
	**UpVermillion**	6vs6	0.500	0.200	7.033	6.382	0.790	1.580	0.652
	**LowVermillion**	6vs6	1.700	1.320	7.350	6.973	1.040	1.080	0.377
	***Mean:***		0.940					1.090	0.330
**DS**	**Upper3**	6vs6	1.000	0.630	59.412	58.917	0.630	0.700	0.500
	**Middle3**	6vs6	0.600	0.450	54.683	54.683	0.270	0.500	0.000
	**Lower3**	6vs6	0.900	0.600	60.150	58.142	1.130	1.970	2.008 *
	**InterPupil**	6vs6	0.600	0.300	62.633	63.052	0.900	3.110 *	−0.418
	**EndoCanth**	6vs6	0.800	0.650	33.867	31.847	1.270	3.140 *	2.020 *
	**ExoCanth**	6vs6	0.300	0.300	86.650	88.348	1.320	3.820 *	−1.698 *
	**AlarBase**	6vs6	0.600	0.520	25.650	25.617	0.120	0.200	0.033
	**AlarWidth**	6vs6	0.500	0.200	32.750	32.597	0.280	0.320	0.153
	**Cheilon**	6vs6	0.600	0.370	49.183	49.183	0.560	1.450	0.000
	**UpLipHeight**	6vs6	1.500	0.680	19.550	19.517	0.250	0.400	0.033
	**Philtrum**	6vs6	0.700	0.330	14.317	13.868	0.840	1.570	0.448
	**ChinHeight**	6vs6	0.400	0.330	41.000	40.533	0.520	0.600	0.467
	**UpVermillion**	6vs6	0.600	0.530	7.517	7.382	1.090	1.280	0.135
	**LowVermillion**	6vs6	0.300	0.150	7.250	6.620	0.930	0.980	0.630
	***Mean:***		0.670					1.430	0.310

Footnote: Each measurement was performed six times under both DA and DiA (“6 vs. 6”). Values marked with an asterisk (*) indicate increased variability or a larger difference between DA and DiA, signifying reduced agreement between the two measurement conditions.

**Table 4:** IntraClass Correlation Coefficient Single measures for Intra-observer reliability.

Group	ICC	CI Lower	CI Upper	* p *
**DA Consultant**	0.999	0.998	1.000	<0.001
**DiA Consultant**	0.999	0.997	1.000	<0.001
**DA + DiA Consultant**	0.999	0.997	0.999	<0.001
**DA resident**	1.000	0.999	1.000	<0.001
**DiA resident**	1.000	0.999	1.000	<0.001
**DA + DiA resident**	0.999	0.999	1.000	<0.001
**DA student**	1.000	1.000	1.000	<0.001
**DiA student**	0.999	0.998	1.000	<0.001
**DA + DiA student**	0.999	0.998	1.000	<0.001

**Table 5:** Intraclass Correlation Coefficients Average measures for inter-observer reliability.

Direct Measurements	ICC	CI Lower	CI Upper	* p *
Consultant vs. resident	1.000	1.000	1.000	<0.001
Consultant vs. student	1.000	1.000	1.000	<0.001
Resident vs. student	1.000	1.000	1.000	<0.001
**Digital measurements**	**ICC**	**CI lower**	**CI upper**	***p***
Consultant vs. resident	1.000	1.000	1.000	<0.001
Consultant vs. student	1.000	1.000	1.000	<0.001
Resident vs. student	1.000	1.000	1.000	<0.001

## Data Availability

The data presented in this study are available from the corresponding author upon reasonable request.
